# Dynamic Complexity of Spontaneous BOLD Activity in Alzheimer’s Disease and Mild Cognitive Impairment Using Multiscale Entropy Analysis

**DOI:** 10.3389/fnins.2018.00677

**Published:** 2018-10-01

**Authors:** Yan Niu, Bin Wang, Mengni Zhou, Jiayue Xue, Habib Shapour, Rui Cao, Xiaohong Cui, Jinglong Wu, Jie Xiang

**Affiliations:** ^1^College of Information and Computer, Taiyuan University of Technology, Taiyuan, China; ^2^Department of Radiology, First Hospital of Shanxi Medical University, Taiyuan, China; ^3^Key Laboratory of Biomimetic Robots and Systems, Ministry of Education, Beijing Institute of Technology, Beijing, China; ^4^Graduate School of Natural Science and Technology, Okayama University, Okayama, Japan

**Keywords:** multiscale entropy, Alzheimer’s disease, mild cognitive impairment, blood oxygen level-dependent signals, dynamic complexity

## Abstract

Alzheimer’s disease (AD) is characterized by progressive deterioration of brain function among elderly people. Studies revealed aberrant correlations in spontaneous blood oxygen level-dependent (BOLD) signals in resting-state functional magnetic resonance imaging (rs-fMRI) over a wide range of temporal scales. However, the study of the temporal dynamics of BOLD signals in subjects with AD and mild cognitive impairment (MCI) remains largely unexplored. Multiscale entropy (MSE) analysis is a method for estimating the complexity of finite time series over multiple time scales. In this research, we applied MSE analysis to investigate the abnormal complexity of BOLD signals using the rs-fMRI data from the Alzheimer’s disease neuroimaging initiative (ADNI) database. There were 30 normal controls (NCs), 33 early MCI (EMCI), 32 late MCI (LMCI), and 29 AD patients. Following preprocessing of the BOLD signals, whole-brain MSE maps across six time scales were generated using the Complexity Toolbox. One-way analysis of variance (ANOVA) analysis on the MSE maps of four groups revealed significant differences in the thalamus, insula, lingual gyrus and inferior occipital gyrus, superior frontal gyrus and olfactory cortex, supramarginal gyrus, superior temporal gyrus, and middle temporal gyrus on multiple time scales. Compared with the NC group, MCI and AD patients had significant reductions in the complexity of BOLD signals and AD patients demonstrated lower complexity than that of the MCI subjects. Additionally, the complexity of BOLD signals from the regions of interest (ROIs) was found to be significantly associated with cognitive decline in patient groups on multiple time scales. Consequently, the complexity or MSE of BOLD signals may provide an imaging biomarker of cognitive impairments in MCI and AD.

## Introduction

Functional connectivity (FC) of spontaneous blood oxygen level-dependent (BOLD) signals in functional magnetic resonance imaging (fMRI) has become an important tool for probing brain function changes in normal aging and neurodegenerative diseases. However, relatively few studies have investigated the temporal dynamics of BOLD signals and its relations with pathologic changes in neurophysiology (Sporns et al., 2000; Friston et al., 2003; Wu et al., 2012). As the most complex organ of the human body, the human brain regulates multifaceted actions with billions of neurons and synapses (Fox et al., 2007). Therefore, the BOLD signals possess complex temporal fluctuations, which could be imitated by nonlinear dynamical processes (Soltysik et al., 2004; Stephan et al., 2008; Yan et al., 2017).

Over the past few years, several statistical methods have been applied to quantify the temporal dynamics of physiological systems. A widely used non-linear statistical method is sample entropy (SE) proposed by Richman and Moorman (2000). SE improved approximate entropy (ApEn) proposed by Pincus (1991), by resolving the problem of erratic results due to vector self-matching. Many studies evidenced the effectiveness of SE in the complexity analysis of time series data of biological systems (Sokunbi et al., 2013, 2014). However, recent studies found that neural signals in the brain possess correlations over a wide range of temporal and spatial scales, stemming from long-range interactions (Costa et al., 2005; Peng et al., 2009; Morabito et al., 2012). Therefore, SE may not be adequate to fully capture the complexity of neural signals by only calculating signal entropy on a single scale.

The multiscale entropy (MSE) was proposed (Costa et al., 2002) to investigate the dynamic complexity of a time series across multiple temporal scales. Several studies have demonstrated the efficacy of MSE for quantifying the complexity of BOLD signals in aging (Yang et al., 2013; Smith et al., 2014). Yang et al. (2013) employed MSE analysis to investigate the complexity of BOLD signals between the younger and older groups, and found significant decreases in MSE in older subjects. Smith et al. (2014) explored the effect of healthy aging on the entropy of resting-state fMRI (rs-fMRI) using MSE analysis, and the results revealed enhanced contrast between healthy young and aged volunteers at longer time scales. However, the dynamic complexity of BOLD signals in neurodegenerative diseases across multiple temporal scales remains largely unexplored.

Alzheimer’s disease (AD) is a neurodegenerative disease characterized by progressive deterioration of cognitive and behavioral function (Ballard et al., 2011). Mild cognitive impairment (MCI) is a neurological disorder occurring before the onset of early AD as an intermediate stage at a high risk of developing AD (Petersen et al., 1999; Belleville et al., 2011). A few studies found decreased complexity of BOLD signals in AD by using single-scale entropy analysis (Liu et al., 2013; Wang et al., 2017). However, the complexity alterations of BOLD signals in MCI and AD patients across multiple time scales remain unclear.

We obtained BOLD rs-fMRI data from the Alzheimer’s disease neuroimaging initiative (ADNI^1^) database, including 30 normal control (NC), 33 early MCI (EMCI), 32 late MCI (LMCI), and 29 AD subjects. MSE maps of the four groups across multiple time scales were calculated and the clusters with significant MSE differences were identified. We then examined the relationships between MSE values and scores of cognitive assessments on all time scales. Finally, we investigated the relationship between MSE and gray matter volume (GMV) on all time scales.

## Materials and Methods

### Participants

A total of 124 subjects were selected from ADNI-2 database, including 30 NC subjects (aged 74.18 ± 5.96 years; 19 females; education: 16.8 ± 2.0 years), 33 EMCI subjects (aged 72.01 ± 5.87 years; 16 females; education: 15.5 ± 2.4 years), 32 LMCI subjects (aged 72.57 ± 8.16 years; 13 females; education: 16.5 ± 2.1 years), and 29 AD subjects (aged 72.33 ± 7.26 years; 18 females; education: 16 ± 2.7 years). For each subject, there were cognitive assessments including Mini-Mental State Examination (MMSE), Clinical Dementia Rating (CDR), and Functional Activities Questionnaire (FAQ). **Table 1** summarizes the demographic and clinical characteristics of the participants.

**Table 1:** Demographic and clinical characteristics of the participants.

	NC	EMCI	LMCI	AD	*p*-value
Age (years)	74.18 ± 5.96	72.01 ± 5.87	72.57 ± 8.16	72.33 ± 7.26	0.505
Sex (M/F)	11/19	17/16	19/13	11/18	0.732
Education (years)	16.8 ± 2.0	15.5 ± 2.4	16.5 ± 2.1	16 ± 2.7	0.418
MMSE	28.9 ± 1.7	27.59 ± 2.02	26.96 ± 2.69	21.0 ± 3.5	<0.001
FAQ	0.14 ± 0.44	3.03 ± 4.50	4.07 ± 4.70	15 ± 7.47	<0.001
CDR	0	0.5	0.5	0.84 ± 0.23	<0.001


*Data are given as the mean ± standard deviation (SD).*

*The *p*-values were obtained by one-way ANOVA.*

### Data Acquisition and Data Processing

All subjects went through resting-state BOLD fMRI scans with their eyes closed on a 3.0 T scanner (Philips Medical Systems) using the following parameters: repetition time (TR) = 3000 ms; echo time (TE) = 30 ms; slice thickness = 3.3 mm; flip angle = 80°; slice number = 48, and 140 time points.

Resting-state fMRI data were preprocessed using Statistical Parametric Mapping (SPM12^2^), Data Processing and Analysis for (Resting-State) Brain Imaging (DPABI; Yan et al., 2016) and the rs-fMRI Data Analysis Toolkit (REST 1.8; Song et al., 2011) packages. The following steps were performed: removing the first 10 time points; slice-timing correction; image realignment; normalization to the Montreal Neurological Institute (MNI) space (resampled into 3 mm × 3 mm × 3 mm voxels). The linear trends of time courses were removed, and the effect of nuisance covariates was removed by signal regression using the global signal, the motion parameters, the cerebrospinal fluid (CSF) and white matter (WM) signals. Temporal filtering (0.01 Hz < *f* < 0.2 Hz) was applied. Finally, each voxel time series was standardized to a mean of zero and standard deviation of unity.

The analysis of the GMV was performed according to the voxel-based morphometry (VBM) protocol using DPABI. The VBM procedure involves the segmentation of the original anatomic MRI images in gray matter (GM), WM, and CSF tissues, followed by GM image normalization to templates in stereotactic space to acquire optimized normalization parameters, which were applied to the raw images. Finally, GM images were smoothed using a 6-mm full-width at half-maximum (FWHM) Gaussian kernel.

### MSE Theory

Multiscale entropy is based on the theory of SE over a range of scales and consists of two steps (Costa et al., 2002).

(1) The coarse-graining procedure of time series represents the system dynamics on different scale factors. Given time series {*x_i_,i* = 1,2,…,*N*}, for the time scale *l*, the coarse-grained time series {*y^l^*} is calculated as follows:

$$y_{j}^{l}=\frac{1}{l}\overset{jl}{\underset{i=(j-1)l+1}{\Sigma }}x_{i,}1\leq j\leq N/l$$

The length of new time series is *N/l*. For scale *1*, the new time series corresponds to the original time series.

(2) The SE for each coarse-grained time series is calculated.

Sample entropy (Richman and Moorman, 2000) is calculated as:

$$SE(m,r,N)=-In\frac{P_{m+1}\left(r\right)}{P_{m}\left(r\right)}$$

where *m* is the sequence length of time points to be compared, *r* is the radius of similarity, *N* is the length of the time series, and *P* is the probability that points falling within *r*.

Multiscale entropy consists of a set of SE values under multiple time scales, which reflects the complexity of time series on multiple scales. MSE can be used to compare the complexity of different time series, based on the specific trend of SE changes with scales (e.g., complex time series show constant entropy over various time scales, while random noise shows a marked decrease in entropy at longer time scales; Wang et al., 2018).

### MSE Calculation

We used the Complexity Toolbox^3^ [Laboratory of Functional MRI Technology (LOFT), Department of Neurology, University of Southern California] to calculate MSE of rs-fMRI data.

Three parameter values were set for the calculation of MSE, including pattern length *m*, distance threshold *r*, and time scale *l*. The point to be made is that the *r* value is generally correlated with the standard deviation of the original time series (Lu et al., 2015). Various theoretical and clinical applications have indicated that, *m* = 1 or 2 and *r* = 0.1–0.35 of the standard deviation of the original sequence, provides reasonable statistical validity for calculating SE (Richman and Moorman, 2000). Because no rigorous standard exists for choosing the parameters to calculate SE, prior studies on SE analysis of biomedical signals have shown inconsistent selection of parameters. For example, studies of fMRI used various parameters, including *m* = 1 and *r* = 0.35 (Yang et al., 2013), *m* = 2 and *r* = 0.30 (Smith et al., 2014), *m* = 2 and *r* = 0.15 (Yang et al., 2011). In addition, different parameters were also used in the studies of EEG, including *m* = 2 and *r* = 0.15 (Catarino et al., 2011), *m* = 2 and *r* = 0.25 (Xiang et al., 2015), *m* = 1 and *r* = 0.25 (Escudero et al., 2006). In this study, MSE was calculated for each BOLD time series based on different parameter pairs: (*m* = 2, *r* = 0.15), (*m* = 2, *r* = 0.25), (*m* = 2, *r* = 0.30), (*m* = 2, *r* = 0.35), (*m* = 1, *r* = 0.25), and (*m* = 1, *r* = 0.35) across the range of scales from 1 to 6.

### Statistical Analyses

For every time scale, one-way analysis of variance (ANOVA) was used to assess differences in MSE maps of BOLD signals among four groups (NC, EMCI, LMCI, and AD) using REST 1.8. For multiple comparison corrections, a stringent statistical significance level was employed by setting a voxelwise threshold of *p* < 0.001 and a cluster threshold of *p* < 0.05 with a Gaussian random field (GRF) correction among four groups after adjusting for age, sex, and education differences.

Then, fivefold cross-validation was used for regions of interest (ROIs) analyses. We divided the data into five independent subsets. For each fold, we used one subset for selective analysis after using other four subsets for selection (ANOVA). According to the peak MNI coordinates (*X Y Z*), we extracted the average MSE and GMV by using DPABI toolbox to define ROIs and the radius of the spheres at all scales (8 mm). For each ROI, differences on MSE values among four groups at all scales were compared using ANOVA using Statistical Package for Social Sciences (SPSS 20.0) software. Bonferroni’s *post hoc* pairwise test on ANOVA was performed.

Spearman’s correlation was used to assess the relationship between MSE and MMSE, FAQ, CDR, and GMV for four groups using SPSS 20.0 software.

## Results

### Demographic and Clinical Characteristics

**Table 1** summarizes the demographic and clinical characteristics of the participants. The *p*-values were obtained by one-way ANOVA. The results indicated no difference in age, sex, and education across four groups. Significant differences (*p* < 0.001) among four groups were found on the MMSE, FAQ, and CDR scores.

### Parameter Selection for MSE Calculation

The comparison was made by calculating MSE using six different parameter combinations (*m, r*). All subjects’ MSE maps were calculated across time scales from 1 to 6. We performed the one-way ANOVA on MSE maps of four groups on every time scale. Based on the final results, the findings using *m* = 2 and *r* = 0.35 as the optimal parameter were mainly reported in this study. Previous study has shown that the accuracy of the calculation results is least dependent on the sequence length *N* when *m* = 2 (Smith et al., 2014). Other results are presented in **Supplementary Data Sheet 1**. For *m* = 2 and *r* = 0.15, four clusters were significantly different among the four groups across multiple time scales on scale 2, scale 3, scale 4, and scale 6 (**Supplementary Table S1** and **Supplementary Figure S1**). Five clusters were found on scale 2, scale 4, and scale 6 when *m* = 2 and *r* = 0.25 (**Supplementary Table S2** and **Supplementary Figure S2**). For *m* = 2, *r* = 0.30 and *m* = 2, *r* = 0.35, similar results were found and nine clusters showed significant differences on scale 2, scale 4, scale 5, and scale 6 (**Supplementary Table S3** and **Supplementary Figure S3**). For *m* = 1, *r* = 0.25 and *m* = 1, *r* = 0.35, however, only one consistent cluster was found (left middle occipital gyrus) on scale 1 and no difference was found on the rest scales (**Supplementary Tables S4**, **S5** and **Supplementary Figures S4**, **S5**).

### Significant Differences on MSE Among the Four Groups

**FIGURE 1:**
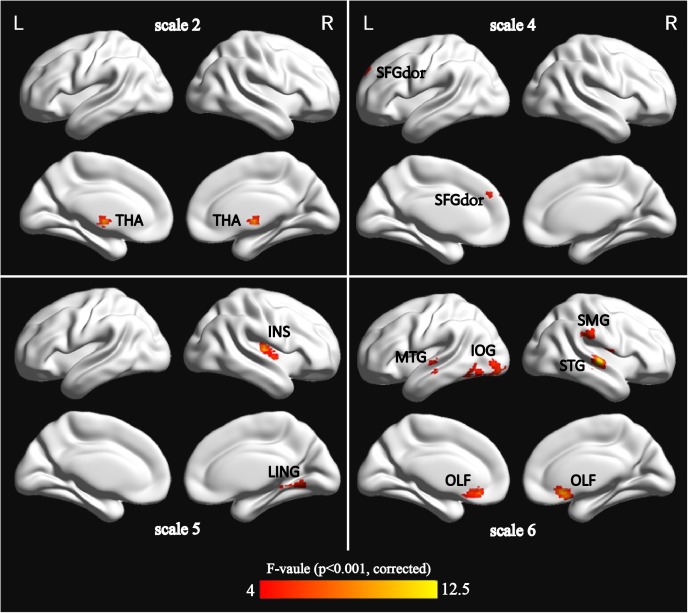
Surface-rendered images showed the differences between the control and patient groups after adjusting for age, sex, and education. The regions showed significantly different brain regions among the four groups on scale 2, scale 4, scale 5, and scale 6. See **Table 2** for a complete list of these regions (threshold *p* < 0.001, GRF corrected).

Using *m* = 2 and *r* = 0.35, the result is presented in **Figure 1**. The detailed information is summarized in **Table 2**. Significant differences (*p* < 0.001, GRF correction) were found on the MSE maps among the four groups on scale 2, scale 4, scale 5, and scale 6. We found no significant difference on scale 1 and scale 3. On scale 2, one cluster was found: right thalamus (THA.R). On scale 4, one cluster was found: left superior frontal gyrus (SFGdor.L). We found two clusters on scale 5: right lingual gyrus (LING.R) and right insula (INS.R). For the scale 6, five clusters were found: right superior temporal gyrus (STG.R), left middle temporal gyrus (MTG.L), right olfactory cortex (OLF.R), left inferior occipital gyrus (IOG.L), and right supramarginal gyrus (SMG.R).

**Table 2:** Characteristics of the brain regions those were significantly different among the four groups across multiple time scales.

Scale	Brain Region	AAL.Abbr	Peak MNI (*X*, *Y*, *Z*)	Cluster voxels	Voxel *F*-value
Scale 2	Thalamus	THA.R	(0, -9, 0)	120	8.817
Scale 4	Superior frontal gyrus	SFGdor.L	(-18, 54, 42)	81	7.043
Scale 5	Lingual gyrus	LING.R	(15, -51, -9)	82	7.948
	Insula	INS.R	(33, -12, 6)	78	9.807
Scale 6	Superior temporal gyrus	STG.R	(60, -18, 0)	153	12.274
	Middle temporal gyrus	MTG.L	(-66, -18, -3)	95	8.258
	Olfactory cortex	OLF.R	(6, 21, -12)	139	10.959
	Inferior occipital gyrus	IOG.L	(-54, -69, -9)	203	7.434
	Supramarginal gyrus	SMG.R	(60, -33, 27)	81	7.177


*The location coordinates are those of the peak significance in each region (*p* < 0.001, GRF corrected).*

**FIGURE 2:**
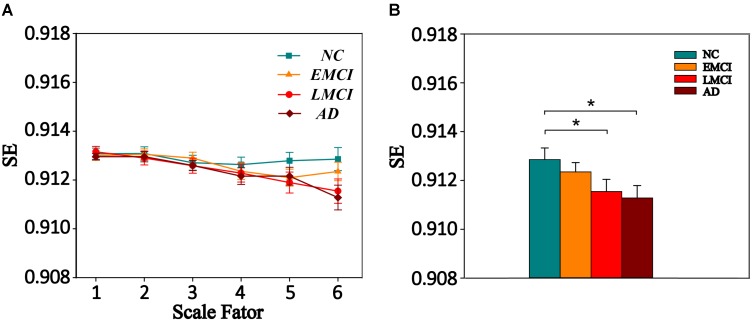
**(A)** MSE curve across scale factor 1–6 in gray matter (GM) for four groups. Each point represents group average SE. **(B)** Mean SE values of GM in the NC, EMCI, LMCI, and AD subjects on scale 6. Significant differences between each pair of the four groups (*p* < 0.05, two-sample *t*-test, uncorrected) are indicated. The error bar represents the standard error of MSE within the group. ^∗^ indicates *p* < 0.05.

We also extracted the mean MSE of whole brain (WB), GM, WM, and CSF using the corresponding masks on all time scales. Then, one-way ANOVA was performed to examine the differences among the four groups. The result is presented in **Supplementary Table S6**. Only GM showed a trend of entropy difference (*F* = 2.283, *p* = 0.083) among four groups on scale 6. **Figure 2** shows the mean entropy curve of GM across the scale of 1–6 for four groups as well as the differences between each pair of the four groups on scale 6 (*p* < 0.05, two-sample *t*-test, uncorrected).

### Time Scales Analysis on MSE From Scale 1 to Scale 6

**FIGURE 3:**
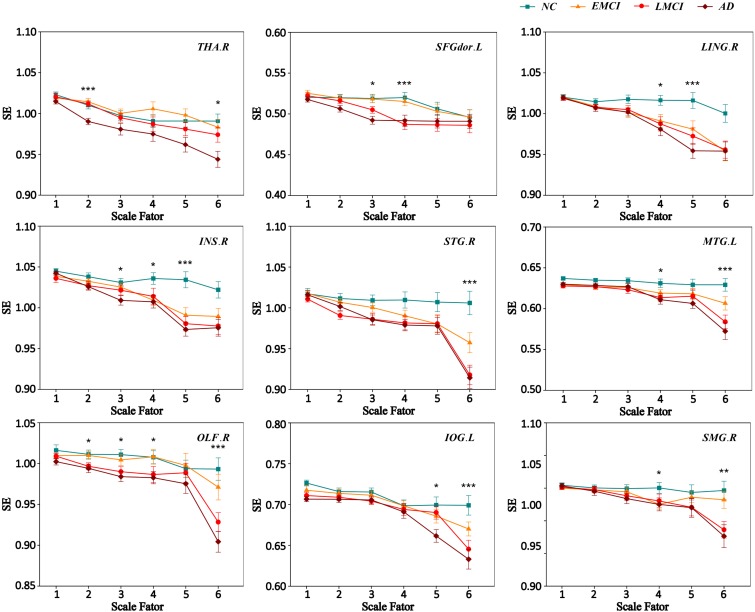
MSE curve across scale factor 1–6 for four groups. Each point represents group average SE. The error bar represents the standard error of MSE within the group. ^∗^ indicates *p* < 0.05. ^∗∗^indicates *p* < 0.05. ^∗∗∗^ indicates *p* < 0.001.

We extracted the average MSE of 9 ROIs over multiple time scales. **Figure 3** displays the MSE curve across scale 1 to 6 among four groups (NC, EMCI, LMCI, and AD) for nine ROIs. Each group exhibited a drop in SE values with increasing scale. SE values on scale 1 showed no difference among the four groups for nine ROIs. For scale 2, there were two ROIs (THA.R and OLF.R) showing significant differences among four groups. SFGdor.L, INS.R, and OLF.R showed significant differences on scale 3. For scale 4, there were six ROIs (SFGdor.L, LING.R, INS.R, MTG.L, OLF.R, and SMG.R) showing significant differences among four groups. There were four ROIs showing significant differences (LING.R, INS.R, and IOG.L) on scale 5. On scale 6, there were six ROIs showing significant differences (THA.R, STG.R, MTG.L, OLF.R, IOG.L, and SMG.R) among four groups. Specifically, OLF.R showed significant differences on four time scales (scale 2, scale 3, scale 4, and scale 6).

### Comparison of MSE Among the Four Groups

**FIGURE 4:**
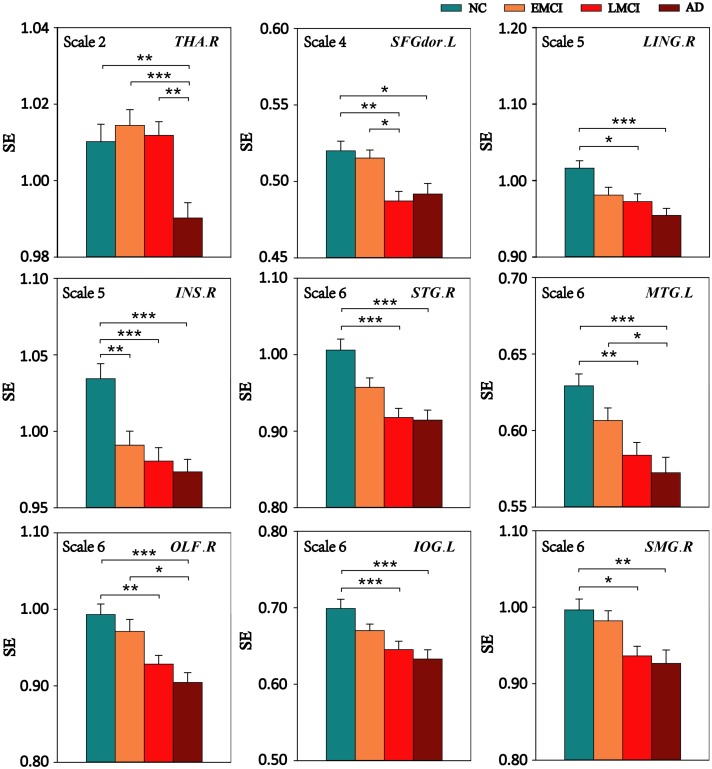
Mean SE values of the nine ROIs in the NC, EMCI, LMCI, and AD subjects on four time scales. Significant differences between pairs of groups after Bonferroni correction (*p* < 0.05) are indicated. The error bar represents the standard error of MSE within the group. ^∗^ indicates *p* < 0.05. ^∗∗^ indicates *p* < 0.01. ^∗∗∗^ indicates *p* < 0.001.

Multiscale entropy values of nine ROIs at all scales were compared among the four groups (NC, EMCI, LMCI, and AD) using ANOVA, and for MSE of ROIs with significant differences among the four groups, Bonferroni’s *post hoc* pairwise test on ANOVA was performed. **Figure 4** displays the comparison of MSE of nine ROIs at different scales between any two groups. The results showed that, compared with NC subjects, patient groups demonstrated reduced complexity. Specifically, the AD group showed lower complexity than the NC group for all ROIs.

Compared with the NC group, the EMCI subjects had significantly reduced MSE of BOLD signals in INS.R. The LMCI subjects showed significantly decreased MSE in eight of the nine ROIs except THA.R. Compared with the EMCI group, the LMCI group showed decreased MSE in SFGdor.L while the AD group showed decreased MSE in three ROIs (THA.R, MTG.L, and OLF.R). In addition, THA.R had lower complexity in the AD group than that in the LMCI group.

### Relationships Between MSE and Clinical Measurements

We performed Spearman’s correlations between MSE values and the clinical measurements (MMSE, FAQ, and CDR) in patient groups (MCI and AD). After corrections for multiple comparisons, significant correlations were found.

**FIGURE 5:**
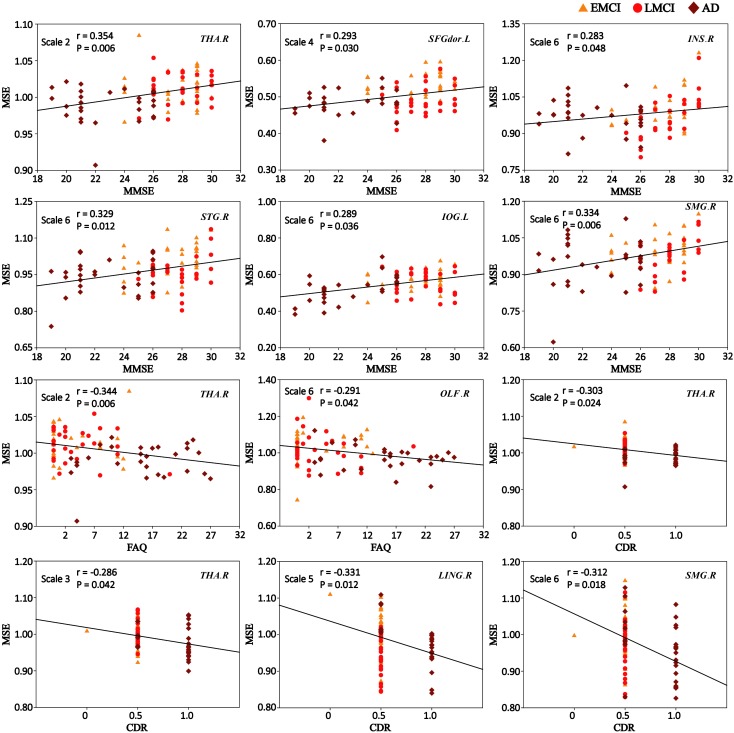
Significant correlations between MSE of blood oxygen level-dependent (BOLD) signals and clinical measurement scores (MMSE, FAQ, and CDR) in patient groups (*p* < 0.05, corrected). *r* is the Spearman correlation coefficient, and *p* indicates the level of statistical significance.

**Figure 5** shows the scatter plots between MSE values of BOLD signals and clinical measurement scores (MMSE, FAQ, and CDR) in patient groups in the significantly correlated brain regions. On scale 2, MMSE was positively correlated with the complexity of BOLD signals in THA.R (*r* = 0.354, *p* = 0.006). SFGdor.L exhibited the significant positive correlation (*r* = 0.293, *p* = 0.030) between the MSE values and MMSE scores on scale 4. The four ROIs (INS.R, STG.R, IOG.L, and SMG.R) exhibited significant positive correlations (*r* > 0.283, *p* < 0.048) between MSE and MMSE scores on scale 6. Some trend correlations were also found (*p* < 0.05, uncorrected) and the results are shown in **Supplementary Table S7**.

As shown in **Figure 5**, the MSE values of THA.R exhibited significant negative correlations (*r* = -0.344, *p* = 0.006) with the FAQ scores on scale 2. OLF.R exhibited significant correlations (*r* = -0.291, *p* = 0.042) between FAQ scores and MSE values of BOLD signals in patient groups on scale 6. SFGdor.L, LING.R, INS.R, and IOG.L exhibited trend correlations (*p* < 0.05, uncorrected) between FAQ scores and MSE values on multiple time scales (**Supplementary Table S8**).

After corrections for multiple comparisons, MSE values of THA.R exhibited the significant negative correlation with the CDR scores on scale 2 (*r* = -0.303, *p* = 0.024) and scale 3 (*r* = -0.286, *p* = 0.042). LING.R showed the significant negative correlation between CDR scores and MSE values on scale 5 (*r* = -0.331, *p* = 0.012) and MSE values of SMG.R were negatively correlated with CDR scores on scale 6 (*r* = -0.312, *p* = 0.018; **Figure 5**). In addition, SFGdor.L, INS.R, STG.R, OLF.R, and IOG.L exhibited trend correlations (*p* < 0.05, uncorrected) between CDR scores and MSE values on multiple time scales. **Supplementary Table S9** summarizes the correlation results between CDR scores and MSE values of BOLD signals in patient groups.

We also performed Spearman’s correlations between MSE values and the clinical measurements (MMSE, FAQ, and CDR) for each group (NC, EMCI, LMCI, and AD). After corrections for multiple comparisons, for the NC group, SMG.R exhibited the significant negative correlation (*r* = -0.516, *p* = 0.048) between MSE values and FAQ scores on scale 5. No correlation was found between MSE values and MMSE, FAQ, and CDR scores in the EMCI group on all scales. For the LMCI group, MSE values of STG.R were positively correlated (*r* = 0.512, *p* = 0.030) with MMSE scores and MSE values of MTG.R and IOG.L were negatively correlated (*r* < -0.485, *p* < 0.048) with FAQ scores. STG.R exhibited the significant negative correlation(*r* = -0.488, *p* = 0.048 between MSE values and CDR scores in the AD group. Some trend correlations were also found (*p* < 0.05, uncorrected) and the results are shown in **Supplementary Tables S11**–**S14**.

### Relationships Between MSE and GMV

We extracted the average GMV values of nine ROIs for four groups. Then, we explored the relationships between the MSE and the GMV in patient groups. After corrections for multiple comparisons, no significant correlation was found between the complexity of BOLD signals and GMV values. But the LING.R exhibited trend positive correlations (*r* > 0.209, *p* < 0.043, uncorrected) between the MSE and the GMV in patient groups on four time scales (scale 3, scale 4, scale 5, and scale 6). STG.R exhibited a positive correlation (*r* = 0.203, *p* = 0.050, uncorrected) between the MSE and the GMV on scale 6 and MTG.L showed a positive correlation (*r* = 0.235, *p* = 0.023, uncorrected) on scale 5. The results are presented in **Supplementary Table S10**.

Correlation analyses for each group (NC, EMCI, LMCI, and AD) were also performed between the MSE and the GMV on all time scales. After corrections for multiple comparisons, no significant correlation was found between the complexity of BOLD signals and GMV values in the NC, EMCI, and LMCI groups. Only SFGdor.L showed the significant positive correlations in the AD group on scale 6. Some brain regions exhibited trend positive correlations (*p* < 0.05, uncorrected) between MSE values and GMV values in each group and the results are presented in **Supplementary Tables S11**–**S14**.

## Discussion

In this study, we employed MSE analysis to assess the complexity of BOLD activity in AD and MCI patients from scale 1 to 6. We discovered that the spontaneous BOLD signals of nine clusters had significant differences among four groups on four time scales. The significant MSE differences were mainly detected in the occipital, frontal, temporal, limbic, and parietal lobes, which were significantly correlated with clinical measurements in patient groups from scale 2 to 6. These results suggest that the complexity analyses using MSE of BOLD signals can provide information on the temporal dynamics of neural signals across multiple scales that are relevant to the cognitive impairments in MCI and AD.

### The MSE Differences Among Four Groups on Multiple Time Scales

This study found that MSE of BOLD activity exhibited significant contrasts among four groups on four time scales (scale 2, scale 4, scale 5, and scale 6; **Figure 1**), mainly distributed in the occipital lobe (IOG.L and LING.R), frontal lobe (SFGdor.L and OLF.R), parietal lobe (SMG.R), temporal lobe (STG.R and MTG.L), limbic lobe (INS.R), and the subcortical region (THA.R). In the MSE analysis for nine ROIs over all time scales, we found that three ROIs (SFGdor.L, INS.R, and OLF.R) had significant differences on scale 3 (**Figure 3**). This means that more useful information was found on multiple time scales. This is consistent with previous reports using MSE analysis on rs-fMRI and EEG signals that detected differences in entropy on multiple time scales (Mizuno et al., 2010; Liu et al., 2013; Yang et al., 2013; Mcbride et al., 2014; Smith et al., 2014; Michalopoulos and Bourbakis, 2017). Particularly, as **Figure 1** demonstrates, five clusters showed significant differences on scale 6. More significant differences were found among the four groups with increasing scales. As can be seen from **Figure 3**, six ROIs showed prominent differences among the four groups on scale 4 and 6. Based on the mechanism of MSE analysis, at the shortest scale, the entropy is dominated by the high frequency fluctuations from random noise (Wang et al., 2018). By filtering out these random fluctuations, the contrast in entropy becomes larger at longer time scales (Smith et al., 2014).

In this study, each of the nine ROIs was observed on a single scale. In the process of calculating MSE, the key step is to coarse-grain the time series to reflect the system dynamics on different time scales, which means that, MSE mainly calculates the complexity of high frequencies at low scales, while the complexity of low frequencies is calculated at large scales. Our results showed that different brain regions displayed differences at different frequencies. Consisted with our result, Wang et al. (2018) investigated the neurophysiological underpinnings of complexity (MSE) of fMRI signals and their relations to FC and the results showed that the associations between MSE and FC were dependent on the temporal scales or frequencies It has been proposed that each frequency band is generated by different mechanisms and relates to different physiological functions, higher frequency oscillations are confined to a small neuronal space, whereas lower frequencies may reflect long-range interactions (Buzsáki and Draguhn, 2004; Zuo et al., 2010). More recently, studies on rs-fMRI have hypothesized that frequency-dependent effects in different brain regions which reflect different synaptic and functional characteristics that are affected by the progression of cognitive impairment (He et al., 2010; Yu et al., 2013; Wang et al., 2016; Zhou et al., 2016). Hence, we propose that the observed complexity changes on different time scales might represent different region or network-dependent neuropathophysiological mechanisms in MCI and AD.

We also analyzed the complexity of WB, GM, WM, and CSF on all time scales. Only GM showed a trend of entropy difference (*F* = 2.283, *p* = 0.083) among four groups on scale 6. Many studies on the complexity analysis of rs-fMRI data found global complexity differences in aging (Yang et al., 2013; Sokunbi et al., 2015) and AD (Liu et al., 2013; Wang et al., 2017). Smith et al. (2014) found greater age-related decline in average GM complexity of rs-fMRI at longer time scales, and Liu et al. (2013) found mean complexity of rs-fMRI in GM and WM decreased with normal aging. Thus, the complexity of global brain activity may decrease with age. For AD-related cognitive decline, Liu et al. (2013) found that mean ApEn of GM showed a significant positive correlation with MMSE scores in the cohort of familial AD subjects, and Wang et al. (2017) found significant differences (*p* < 0.05) in permutation entropy (PE) of GM and WM across the four groups of ADNI data. Possibly due to differences in data samples and complexity analysis methods, the MSE analysis used in this study was only able to reveal a trend of entropy difference (*p* = 0.083) among four groups in GM as well as decreased complexity in the AD and LMCI groups compared to that of the NC group at the statistical threshold of uncorrected *p* < 0.05. In contrast, we found highly significant MSE differences (*p* < 0.001, GRF corrected) in several brain regions on multiple time scales. This is not surprising as the pathological process of AD first affects the network of temporal, frontal, and parietal regions before progressing to the whole GM and brain level. Different complexity analyses may have different sensitivities in detecting global and regional changes of neural complexity with AD progression. This question awaits further investigation in future studies.

### Decreased Complexity and Cognitive Decline in Patient Groups

Using the *post hoc* pairwise test on ANOVA, reduced complexity in the AD group was detected in all ROIs compared with the NC group (*p* < 0.05, Bonferroni corrected). In addition, MSE also showed strong sensitivity in differentiating NC from EMCI (one ROI), NC from LMCI (eight ROIs), EMCI from LMCI (one ROI), EMCI from AD (three ROIs), and LMCI from AD (one ROI). As can be seen from **Figure 4**, the complexity of BOLD signals in most ROIs showed a gradually decline from NC to EMCI to LMCI and to AD. Previous complexity studies of fMRI signals also consistently reported reduced complexity in AD patients compared to matched control subjects (Liu et al., 2013; Wang et al., 2017). Liu et al. (2013) reported decreased complexities in STG, MTG, and SMG in familial AD. Some of brain regions, such as SFGdor, MTG, and IOG, were also reported in our previous study using PE method to analyze the complexity of the same ADNI dataset (Wang et al., 2017).

We performed correlation analyses between complexity of BOLD signals in these brain regions with significant MSE differences and cognitive function scores (MMSE, FAQ, and CDR). These three clinical measurements provide quantitative assessments of cognitive function and are widely used (Ciesielska et al., 2016; Kaur et al., 2016; Kim et al., 2017). Higher scores of MMSE indicate higher aptitude of cognition; low functional performance is related to higher FAQ scores and the presence of dementia is indicated by higher CDR scores. Our correlation results showed that the average MSE of some brain regions was significantly positively correlated with the MMSE scores and significantly negatively correlated with FAQ scores and CDR scores in patient groups (*p* < 0.05, corrected). This means that lower MMSE and higher FAQ and CDR scores were observed in MCI and AD patients who exhibited lower MSE in some brain regions. Particularly, THA.R exhibited significant correlations between MSE values and three clinical measurement scores (MMSE, FAQ, and CDR) on scale 2. The MSE values of SMG.R showed significant correlations with the MMSE and CDR scores on scale 6. Previous fMRI studies suggested that THA and SMG are closely related to cognitive dysfunction in healthy aging and AD (Mevel et al., 2011; Yang et al., 2013; Xiaoying et al., 2014; Raczek et al., 2017). Studies found that activity in THA is associated with spatial working memory and memory processing (Jankowski et al., 2013; Saalmann and Kastner, 2015; Štillová et al., 2015; Hallock et al., 2016), and SMG is mainly involved in language perception, phonological processing and verbal working memory and processing (Hartwigsen et al., 2010; Kheradmand et al., 2013; Deschamps et al., 2014). In addition, as can be seen from **Figure 5**, significant correlations between MSE and cognitive measurements were dependent on the temporal scales. For example, we observed THA.R showed associations between complexity and MMSE at high temporal frequencies, and SMG.R exhibited significant correlations at low temporal frequencies. The results showed that different brain regions displayed correlations at different frequencies and once again corroborated the MSE theory that high and low temporal frequencies may represent region or network-dependent different neuropathophysiological mechanisms (Buzsáki and Draguhn, 2004; Zuo et al., 2010).

### Potential Physiological Underpinnings of Altered Complexity in Patient Groups

It has been suggested that physiological diseases are associated with a loss of complexity in healthy systems (Lipsitz, 2004; Pincus, 2010). AD is a neurodegenerative disorder characterized by dementia and cognitive decline (Querfurth and Laferla, 2010). The brain regions that we found to have reduced complexity play important roles for cognitive functions. For example, the lingual gyrus is believed to play a role in the analysis of logical conditions and encoding visual memories. The superior temporal gyrus is involved in social cognition processes and middle temporal gyrus is mainly involved in the recognition of known faces and episodic memory (Bigler et al., 2007; Acheson and Hagoort, 2013). Some fMRI experiments have found proof that the superior frontal gyrus is involved in self-awareness, sensory system, and social cognitive processes (Goldberg et al., 2006). The altered complexity of these brain regions in patient groups may be associated with deterioration of brain function in these important networks.

Further, AD is characterized by the presence of neuritic plaques and neurofibrillary tangles, accompanied by widespread cortical neuronal loss, and loss of connections between brain systems (Sankari, 2010). This may degrade cortical and sub-cortical connections, leading to cognitive and behavioral disturbances. Many studies have reported that disrupted FC in the AD group in THA, SFGdor, INS, STG, MTG, IOG, and SMG (Zhang et al., 2009; Wang et al., 2010; Dennis and Thompson, 2014). Thus, this degeneration of both local and long-range connections disrupts the functional coherence of brain activation, decreasing the complexity of spontaneous brain activity.

In addition, we examined the relationships between MSE and GMV in patient groups. After corrections for multiple comparisons, no significant correlation was found between the complexity of BOLD signals and GMV. But LING.R, STG.R, and MTG.L exhibited trend positive correlations (*p* < 0.05, uncorrected) between the MSE and the GMV in patient groups. Many studies have also reported GM atrophy in these brain regions in MCI and AD (Busatto et al., 2003; Karas et al., 2004; Guo et al., 2010; Möller et al., 2013). In our previous study, we also found that the complexity of these brain regions was related to GMV and was associated with glucose metabolism (Wang et al., 2017). More pathologies of AD may lead to lower complexity of brain regions still requires further study.

### Comparison of SE, PE, MSE, and Multiscale PE

Sample entropy solved the problem of vector self-matching in the ApEn defined by the Heaviside function and has been widely used (Pincus, 1991; Richman and Moorman, 2000). PE is different from SE, as PE calculates the probability of a symbolic sequence of points in the phase space and the entropy value in the form of Shannon information entropy (Bandt and Pompe, 2002). Many researchers prefer to use SE and PE to study the complexity of time series and obtain findings on a single scale (Sokunbi et al., 2013; Berger et al., 2017; Wang et al., 2017; Aktaruzzaman, 2018). Compared with PE and SE, MSE and multiscale PE (MPE) investigate the dynamic complexity of time series data across multiple temporal scales, not only at the original time scale of 1 (Costa et al., 2002; Aziz and Arif, 2005; Ouyang et al., 2013).

In this study, we found significant complexity differences among four groups on multiple temporal scales, especially on longer time scales, due to MSE’s capability to average out short time scale fluctuations (Smith et al., 2014; Yan et al., 2017). Thus, compared with SE, researchers performed MSE for complexity analysis obtained richer and more comprehensive information in aging and neurodegenerative diseases (Humeauheurtier, 2016; Shang, 2017). Our previous research investigated the abnormal complexity of BOLD signals in MCI and AD patients using PE analysis (Wang et al., 2017). Then, we also applied MPE to the same dataset, but no significant difference was found on longer scales (*p* < 0.005, GRF correction). Some studies demonstrated that PE had better anti-noise performance and thus, compared with SE, we got supplementary information in detecting differences among four groups on scale 1 (Bandt and Pompe, 2002; Nicolaou and Georgiou, 2012; Wang et al., 2017). The coarse-grained procedures in MPE with large scale factors may result in short data length, while PE requires more time points to contain more states of the reconstructed sequence (Bandt and Pompe, 2002). This may be the reason that we did not detect the significant PE difference at longer time scales. As a consequence, for our dataset, MPE had better performance at short time scales, while MSE could provide more information on multiple time scales. In the future, we will perform and compare SE and PE analysis across multiple time scales on more rs-fMRI datasets to further our understanding on this issue.

### Limitation

A limitation of this study is the short BOLD time series used for MSE analysis which may lead to potentially erratic entropy estimation (Costa et al., 2002; Yang et al., 2013). In this study, we performed the parameter selection for MSE calculation by using 6 different parameter pairs based on previous studies, not all of the parameter pairs. The results of *m* = 2 and *r* = 0.35 were mainly reported in this study. The selection of parameters may be related to particular datasets, and different datasets may have different optimal parameters.

## Conclusion

Multiscale entropy is a powerful tool to quantify the nonlinear information of a time series over multiple time scales through the SE algorithm. This study applied MSE analysis to investigate the abnormal complexity of BOLD signals across multiple time scales in MCI and AD patients. Enhanced MSE differences were detected among four groups which were significantly correlated with clinical assessments in patient groups at multiple temporal scales. The MCI and AD patients demonstrated lower complexity than normal controls and AD patients showed lower complexity than MCI. These findings indicate that MSE of spontaneous BOLD signals may provide an imaging marker of cognitive impairment in MCI and AD.

## Alzheimer’s Disease Neuroimaging Initiative

The data used in preparation of this article were obtained from the Alzheimer’s disease neuroimaging initiative (ADNI) database adni.loni.usc.edu. As such, the investigators within the ADNI contributed to the design and implementation of the ADNI and/or provided data but did not participate in analysis or writing of this report. A complete listing of the ADNI investigators can be found at http://adni.loni.usc.edu/wpcontent/uploads/how_to_apply/ADNI_Acknowledgement_List.pdf.

## Author Contributions

YN performed the experiment and completed the manuscript. BW, MZ, JX, HS, RC, and XC provided suggestions for this study. JX provided the guidance throughout the study.

## Conflict of Interest Statement

The authors declare that the research was conducted in the absence of any commercial or financial relationships that could be construed as a potential conflict of interest.

## Footnotes

1 http://adni.loni.usc.edu/

2 http://www.fil.ion.ucl.ac.uk/spm

3 http://loft-lab.org/index-5.html
