# Supplementary material for: Dynamic Complexity of Spontaneous BOLD Activity in Alzheimer’s Disease and Mild Cognitive Impairment Using Multiscale Entropy Analysis
Source: Front Neurosci. 2018 Oct 1;12:677. doi: 10.3389/fnins.2018.00677 (PMC6174248; doi:10.3389/fnins.2018.00677)
Supplement: Supplementary file 1 [file Data_Sheet_1.PDF]

In this study, MSE was calculated for each BOLD time series based on different parameter pairs: (*m* = 2, *r* =0.15), (*m*=2, *r*=0.25), (*m*=2, *r*=0.30), (*m*=2, *r*=0.35), (*m*=1, *r*=0.25) and (*m*=1, *r*=0.35) across the range of scales from 1 to 6. The findings using m = 2 and r = 0.35 as the optimal parameter were mainly reported in the manuscript. Other results are as follows:

Table S1. Characteristics of the brain regions those were significantly different among the four groups across multiple time scales when *m*=2 and *r*=0.15

Scale	Brain Region	AAL.Abbr	Peak MNI (X, Y, Z)	Cluster voxels	Voxel F value
Scale 2	Middle Occipital Gyrus	MOG.L	(-51, -75, 0)	31	10.026
Scale 3	Middle Frontal Gyrus	MFG.R	(48, 24, 33)	39	8.681
Scale 4	Lingual Gyrus	LING.L	(-6, -93, -18)	33	8.199
Scale 6	Middle Frontal Gyrus	MFG.R	(45, 21, 27)	56	8.587

The location coordinates are those of the peak significance in each region (*p*<0.001, GRF corrected).

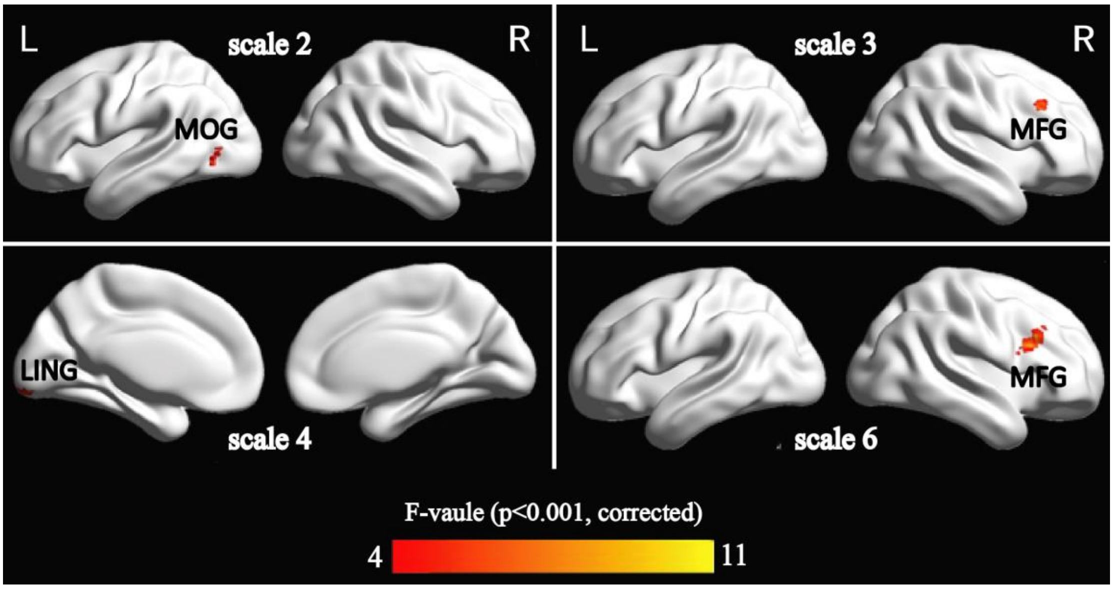

Figure S1. Surface-rendered images showed the differences between the control and patient groups after adjusting for age, sex and education. The regions showed significantly different brain regions among the four groups on scale 2, scale 3, scale 4 and scale 6. See Table S1 for a complete list of these regions (threshold *p*<0.001, GRF corrected).

Table S2. Characteristics of the brain regions those were significantly different among the four groups across multiple time scales when m=2 and r=0.25

Scale	Brain Region	AAL.Abbr	Peak MNI (X, Y, Z)	Cluster voxels	Voxel F value
Scale 2	Thalamus	THA.R	(-3, -9, -3)	62	11.234
Scale 4	Superior Frontal Medial Gyrus	SFGmed.L	(-12, 54, 45)	54	7.709
Scale 6	Middle Occipital Gyrus	MOG.L	(-36, -93, -6)	70	8.197
	Superior Temporal Gyrus	STG.R	(60, -15, 0)	66	10.472
	Olfactory Cortex	OLF_R	(6, 21, -12)	65	8.169

The location coordinates are those of the peak significance in each region (p<0.001, GRF corrected).

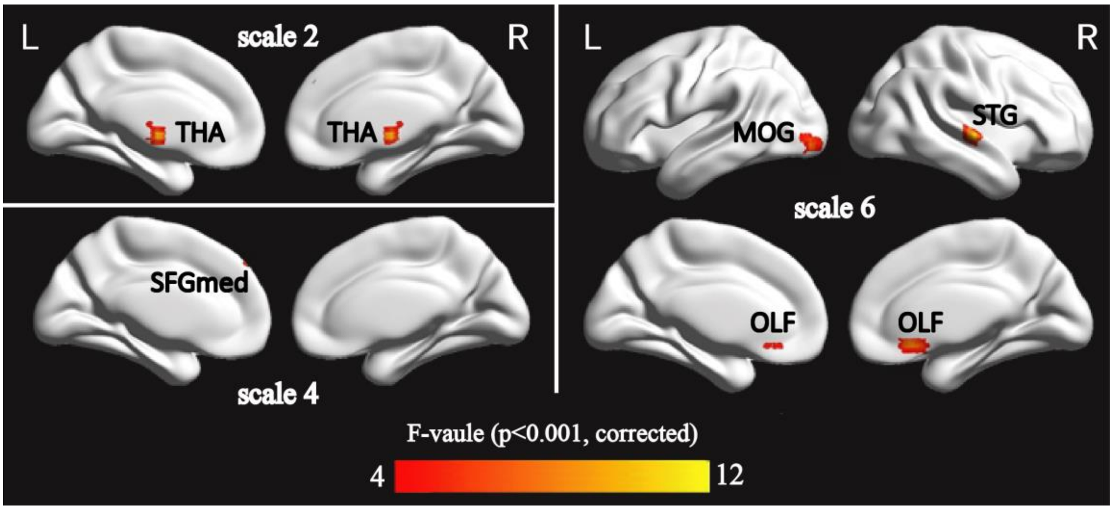

Figure S2. Surface-rendered images showed the differences between the control and patient groups after adjusting for age, sex and education. The regions showed significantly different brain regions among the four groups on scale 2, scale 4 and scale 6. See Table S2 for a complete list of these regions (threshold p<0.001, GRF corrected).

Table S3. Characteristics of the brain regions those were significantly different among the four groups across multiple time scales when m=2 and r=0.30

Scale	Brain Region	AAL.Abbr	Peak MNI (X, Y, Z)	Cluster voxels	Voxel F value
Scale 2	Thalamus	THA.R	(0, -9, 0)	55	8.919
Scale 4	Superior Frontal Gyrus	SFGdor.L	(-12, 51, 27)	64	7.395
Scale 5	Lingual Gyrus	LING.R	(15, -48, -12)	86	9.134
	Insula	INS.R	(36, -15, 9)	81	10.170
Scale 6	Superior Temporal Gyrus	STG.R	(60, -18, -3)	132	11.227
	Middle Temporal Gyrus	MTG.L	(-42, -9,24)	120	9.266
	Olfactory Cortex	OLF.R	(3, 21, -12)	113	10.216
	Inferior Occipital Gyrus	IOG.L	(-36,-93,-9)	110	9.298
	Fusiform Gyrus	FFG.L	(-39, -63, -15)	88	7.520

The location coordinates are those of the peak significance in each region (p<0.001, GRF corrected).

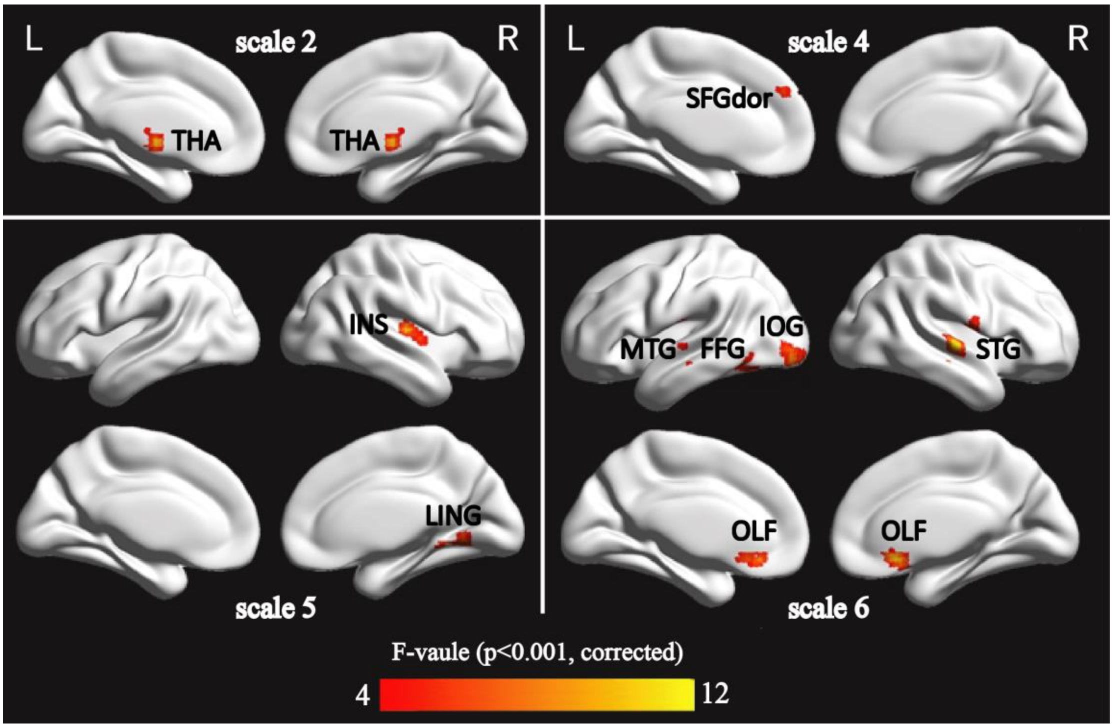

Figure S3. Surface-rendered images showed the differences between the control and patient groups after adjusting for age, sex and education. The regions showed significantly different brain regions among the four groups on scale 2, scale 4, scale 5 and scale 6. See Table S3 for a complete list of these regions (threshold *p*<0.001, GRF corrected).

Table S4. Characteristics of the brain regions those were significantly different among the four groups across multiple time scales when *m*=1 and *r*=0.25

Scale	Brain Region	AAL.Abbr	Peak MNI (X, Y, Z)	Cluster voxels	Voxel F value
Scale 1	Middle Occipital Gyrus	MOG.L	(-30, -90, 0)	63	8.174

The location coordinates are those of the peak significance in each region (p<0.001, GRF corrected).

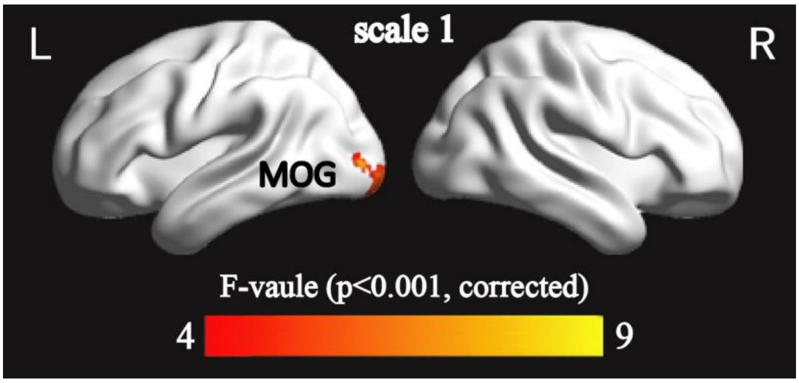

Figure S4. Surface-rendered images showed the differences between the control and patient groups after adjusting for age, sex and education. The regions showed significantly different brain regions among the four groups on scale1. See Table S4 for a complete list of these regions (threshold p<0.001, GRF corrected).

Table S5. Characteristics of the brain regions those were significantly different among the four groups across multiple time scales when m=1 and r=0.35

Scale	Brain Region	AAL.Abbr	Peak MNI (X, Y, Z)	Cluster voxels	Voxel F value
Scale 1	Middle Occipital Gyrus	MOG.L	(-30, -93, 0)	66	7.657

The location coordinates are those of the peak significance in each region (p<0.001, GRF corrected).

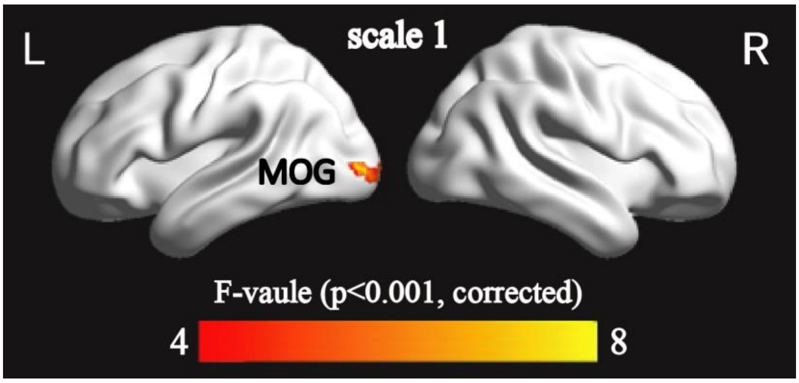

Figure S5. Surface-rendered images showed the differences between the control and patient groups after adjusting for age, sex and education. The regions showed significantly different brain regions among the four groups on scale 1. See Table S5 for a complete list of these regions (threshold p<0.001, GRF corrected).

Table S6. Results of the one-way ANOVA in the mean SE of the whole brain (WB), gray matter (GM), white matter (WM), and cerebral spinal fluid (CSF) among four groups on all time scales.* indicates $p<0.1$.

	WB(F,*p*)	GM (F,*p*)	WM(F,*p*)	CSF(F,*p*)
Scale 1	0.125, 0.945	0.190, 0.903	0.732, 0.535	0.270, 0.847
Scale 2	0.578, 0.630	0.115, 0.951	0.158, 0.924	0.778, 0.508
Scale 3	0.439, 0.725	0.281, 0.839	0.455, 0.714	1.305, 0.276
Scale 4	0.826, 0.482	0.315, 0.814	0.247, 0.863	1.843, 0.143
Scale 5	0.740, 0.530	0.999, 0.395	0.175, 0.913	1.183, 0.319
Scale 6	0.331, 0.803	**2.283, 0.083 ***	0.734, 0.534	2.063, 0.109

Table S7. Results of the correlation analyses between the MSE values and the MMSE in patient groups (MCI and AD).In the table, *r* is the Spearman correlation coefficient, and *p* indicates the level of statistical significance. Significant correlations are indicated in **bold**.

Brain region Abbr.	Scale 2 (*r*, *p*)	Scale 3 (*r*, *p*)	Scale 4 (*r*, *p*)	Scale 5 (*r*, *p*)	Scale 6 (*r*, *p*)
THA.R	**0.354, 0.001**	**0.246, 0.020**	**0.220, 0.039**	**0.229, 0.031**	0.144, 0.185
SFGdor.L	**0.248, 0.021**	0.146, 0.172	**0.293, 0.005**	**0.241, 0.023**	0.109, 0.319
LING.R	0.079, 0.459	0.064, 0.549	0.107,0.320	**0.275, 0.010**	0.164, 0.132
INS.R	0.166, 0.119	**0.222, 0.036**	**0.225, 0.034**	**0.245, 0.021**	**0.283, 0.008**
STG.R	0.133,0.214	**0.237, 0.025**	0.164, 0.124	0.086, 0.424	**0.329, 0.002**
MTG.L	0.159,0.136	**0.256, 0.015**	0.145, 0.176	0.090, 0.404	0.110, 0.315
OLF.R	0.179,0.093	0.136, 0.205	0.110, 0.304	0.077, 0.473	**0.244, 0.025**
IOG.L	0.078,0.467	0.084, 0.436	0.014, 0.898	0.040, 0.710	**0.289, 0.006**
SMG.R	0.015,0.557	0.097, 0.367	0.001, 0.991	0.153, 0.153	**0.334, 0.001**

Table S8. Results of the correlation analyses between the MSE values and the FAQ scores in patient groups (MCI and AD).In the table, *r* is the Spearman correlation coefficient, and *p* indicates the level of statistical significance. Significant correlations are indicated in **bold**.

Brain region Abbr.	Scale 2 (*r*, *p*)	Scale 3 (*r*, *p*)	Scale 4 (*r*, *p*)	Scale 5 (*r*, *p*)	Scale 6 (*r*, *p*)
THA.R	**-0.344, 0.001**	-0.159, 0.137	**-0.209, 0.049**	-0.026, 0.812	**-0.210, 0.048**
SFGdor.L	-0.030, 0.780	**-0.252, 0.017**	**-0.259, 0.014**	-0.163, 0.127	-0.147, 0.177
LING.R	-0.126, 0.238	-0.175, 0.105	-0.008, 0.944	**-0.230, 0.030**	-0.091, 0.407
INS.R	-0.034, 0.752	-0.063,0.554	-0.036, 0.735	**-0.242, 0.023**	-0.194, 0.074
STG.R	-0.100, 0.352	-0.088, 0.414	-0.025, 0.817	-0.042, 0.698	-0.019, 0.864
MTG.L	-0.104, 0.331	-0.124, 0.247	-0.124, 0.155	-0.108, 0.314	-0.025, 0.819
OLF.R	-0.155, 0.148	-0.001, 0.995	-0.152, 0.155	-0.081, 0.448	**-0.291, 0.007**
IOG.L	-0.165, 0.121	-0.069, 0.520	-0.111, 0.299	**-0.218, 0.040**	**-0.248, 0.022**
SMG.R	-0.069, 0.518	-0.032, 0.767	-0.056, 0.602	-0.034, 0.749	-0.126, 0.248

Table S9. Results of the correlation analyses between the MSE values and the CDR scores in patient groups (MCI and AD).In the table, *r* is the Spearman correlation coefficient, and *p* indicates the level of statistical significance. Significant correlations are indicated in **bold**.

Brain region Abbr.	Scale 2 (*r*, *p*)	Scale 3 (*r*, *p*)	Scale 4 (*r*, *p*)	Scale 5 (*r*, *p*)	Scale 6 (*r*, *p*)
THA.R	**-0.303, 0.004**	**-0.286, 0.007**	-0.128, 0.232	-0.179, 0.094	-0.171, 0.115
SFGdor.L	-0.090, 0.400	-0.103, 0.337	**-0.209, 0.049**	-0.090, 0.401	-0.151, 0.166
LING.R	-0.159, 0.138	-0.018, 0.870	-0.046, 0.672	**-0.331, 0.002**	-0.174, 0.110
INS.R	-0.127, 0.236	**-0.209, 0.050**	**-0.220, 0.042**	**-0.265, 0.014**	**-0.277, 0.010**
STG.R	-0.024, 0.823	-0.144, 0.178	-0.113, 0.293	-0.022, 0.836	**-0.275, 0.010**
MTG.L	-0.036, 0.741	-0.043, 0.691	-0.021, 0.845	-0.076, 0.479	-0.025, 0.821
OLF.R	-0.141, 0.187	-0.042, 0.696	-0.082, 0.444	0.007, 0.951	**-0.241, 0.025**
IOG.L	-0.106, 0.323	-0.018, 0.870	-0.055, 0.612	**-0.219, 0.046**	-0.199, 0.060
SMG.R	-0.063, 0.557	-0.098, 0.362	0.009, 0.935	-0.026, 0.811	**-0.312, 0.003**

Table S10. Results of the correlation analyses between the MSE values and gray matter volume (GMV) values in patient groups (MCI and AD). In the table, *r* is the Spearman correlation coefficient, and *p* indicates the level of statistical significance. Significant correlations are indicated in **bold**.

Brain region Abbr.	Scale 3 (*r*, *p*)	Scale 4 (*r*, *p*)	Scale 5 (*r*, *p*)	Scale 6 (*r*, *p*)
THA.R	-0.011, 0.914	-0.043, 0.682	-0.085, 0.417	0.066, 0.530
SFGdor.L	0.002, 0.988	0.038, 0.714	0.099, 0.343	0.149, 0.153
LING.R	**0.234, 0.023**	**0.223, 0.030**	**0.213, 0.039**	**0.209, 0.043**
INS.R	0.118, 0.259	0.108, 0.302	0.027, 0.797	0.086, 0.409
STG.R	0.141, 0.175	-0.056, 0.589	0.043, 0.677	**0.203, 0.050**
MTG.L	0.168, 0.106	0.128, 0.218	**0.235, 0.023**	0.145, 0.162
OLF.R	0.001, 0.996	0.056, 0.594	-0.038, 0.717	0.028, 0.785
IOG.L	-0.074, 0.478	0.038, 0.715	0.015, 0.888	0.003, 0.975
SMG.R	0.029, 0.778	0.133, 0.203	0.026, 0.801	0.078, 0.455

Table S11. Results of the correlation analyses between the MSE values and the MMSE, FAQ and GMV in the NC group. In the table, *r* is the Spearman correlation coefficient, and *p* indicates the level of statistical significance. Significant correlations are indicated in **bold**.

	Brain region Abbr.	Scale 2 (*r*, *p*)	Scale 3 (*r*, *p*)	Scale 4 (*r*, *p*)	Scale 5 (*r*, *p*)	Scale 6 (*r*, *p*)
MMSE	OLF.R	0.167,0.404	**0.399,0.039**	0.340,0.083	0.146,0.468	**0.440,0.022**
FAQ	THA.R	--0.143,0.476	-0.120,0.550	-0.255,0.200	**-0.444,0.020**	-0.224,0.262
	SFGdor.L	0.098,0.625	-0.186,0.352	-0.039,0.846	**-0.410,0.034**	-0.266,0.180
	STG.R	**-0.400,0.039**	-0.065,0.746	-0.080,0.692	-0.004,0.985	-0.064,0.752
	SMG.R	-0.065,0.746	-0.110,0.586	-0.269,0.176	**-0.516,0.006**	-0.259,0.193
GMV	INS.R	**0.394,0.035**	0.148,0.444	0.128,0.508	0.019,0.921	0.220,0.251
	MTG.L	0.055,0.776	0.025,0.897	0.197,0.306	**0.455,0.013**	0.121,0.533
	IOG.L	0.285,0.134	0.162,0.401	**0.422,0.023**	**0.415,0.025**	0.189,0.327
	SMG.R	0.039,0.839	0.115,0.552	**0.430,0.020**	0.166,0.391	-0.054,0.780

Table S12. Results of the correlation analyses between the MSE values and GMV in the EMCI group. In the table, *r* is the Spearman correlation coefficient, and *p* indicates the level of statistical significance. Significant correlations are indicated in **bold**.

	Brain region Abbr.	Scale 2 (*r*, *p*)	Scale 3 (*r*, *p*)	Scale 4 (*r*, *p*)	Scale 5 (*r*, *p*)	Scale 6 (*r*, *p*)
GMV	MTG.L	-0.033,0.856	**0.363,0.038**	-0.202,0.261	0.175,0.329	0.246,0.167
	IOG.L	0.214,0.233	0.115,0.523	-0.038,0.833	-0.090,0.620	**0.397,0.022**
	SMG.R	0.021,0.906	0.053,0.768	0.176,0.328	-0.001,0.997	**0.358,0.041**

Table S13. Results of the correlation analyses between the MSE values and MMSE, FAQ and GMV in the LMCI group. In the table, *r* is the Spearman correlation coefficient, and *p* indicates the level of statistical significance. Significant correlations are indicated in **bold**.

	Brain region Abbr.	Scale 2 (*r*, *p*)	Scale 3 (*r*, *p*)	Scale 4 (*r*, *p*)	Scale 5 (*r*, *p*)	Scale 6 (*r*, *p*)
MMSE	LING.R	-0.019,0.924	0.090,0.641	0.092,0.637	**0.393,0.035**	0.220,0.251
	STG.R	0.311,0.100	**0.383,0.040**	-0.072,0.709	0.174,0.368	**0.512,0.005**
	MTG.L	0.112,0.564	0.293,0.123	0.211,0.272	0.156,0.420	**0.383,0.040**
	IOG.L	0.057,0.770	0.156,0.420	0.080,0.681	**0.413,0.026**	0.340,0.071
FAQ	STG.R	-0.033,0.865	--0.037,0.849	0.057,0.767	**-0.392,0.035**	0.003,0.989
	MTG.L	**-0.385,0.039**	-0.254,0.184	**-0.485,0.008**	-0.242,0.206	-0.279,0.143
	IOG.L	-0.283,0.136	-0.119,0.539	-0.270,0.156	-0.028,0.887	**-0.584,0.001**
GMV	IOG.L	0.071,0.699	0.058,0.753	**0.397,0.025**	**0.433,0.013**	**0.370,0.037**

Table S14. Results of the correlation analyses between the MSE values and MMSE, FAQ, CDR and GMV in the AD group.In the table, *r* is the Spearman correlation coefficient, and *p* indicates the level of statistical significance. Significant correlations are indicated in **bold**.

	Brain region Abbr.	Scale 2 (*r*, *p*)	Scale 3 (*r*, *p*)	Scale 4 (*r*, *p*)	Scale 5 (*r*, *p*)	Scale 6 (*r*, *p*)
MMSE	THA.R	-0.087,0.661	**0.439,0.019**	0.208,0.289	**0.466,0.013**	0.149,0.449
	OLF.R	-0.007,0.971	-0.005,0.981	**0.429,0.023**	0.094,0.635	0.066,0.740
	IOG.L	**0.402,0.034**	0.187,0.342	**0.383,0.044**	0.302,0.118	0.370,0.053
FAQ	STG.R	-0.031,0.874	0.071,0.718	-0.183,0.351	0.060,0.760	**-0.459,0.014**
CDR	INS.R	-0.033,0.867	0.043,0.830	-0.043,0.830	-0.204,0.299	**-0.450,0.016**
	STG.R	-0.043,0.830	-0.099,0.615	-0.355,0.064	-0.071,0.720	**-0.488,0.008**
	MTG.L	-0.241,0.216	-0.213,0.276	-0.128,0.517	**-0.374,0.050**	-0.260,0.181
	OLF.R	-0.024,0.905	-0.204,0.299	**-0.450,0.016**	-0.194,0.322	-0.071,0.720
GMV	SFGdor.L	0.318,0.093	0.114,0.555	-0.072,0.709	-0.009,0.962	**0.490,0.007**
	STG.R	-0.018,0.927	0.244,0.201	0.310,0.101	-0.018,0.925	**0.393,0.035**
